# Design, Implementation, and Verification of High-Accuracy Trapezoidal Dual-Axis Sun Sensors for LEO Satellite Attitude Determination

**DOI:** 10.3390/s26113317

**Published:** 2026-05-23

**Authors:** Mang Ou-Yang, Ching-I Tai, Guan-Yu Huang, Tse-Yu Cheng, Chang-Hsun Liu, Yu-Siou Liu, Jin-Chern Chiou, Chen-Yu Chan, Tung-Yun Hsieh, Chen-Tsung Lin, Ying-Wen Jan, Chih-Hsun Lin, Yung-Jhe Yan

**Affiliations:** 1 Institute of Electrical and Control Engineering, National Yang Ming Chiao Tung University, Hsinchu 300, Taiwan; oym@nycu.edu.tw (M.O.-Y.); gghh711.ee10@nycu.edu.tw (C.-I.T.); nctu0860066.ece08g@nctu.edu.tw (T.-Y.C.); a309512043.ee09@nycu.edu.tw (C.-H.L.); yusiouliu.ee09@nycu.edu.tw (Y.-S.L.); chiou@nycu.edu.tw (J.-C.C.); 2 Institute of Biomedical Engineering, National Yang Ming Chiao Tung University, Hsinchu 300, Taiwan; kay851129.cm08g@nctu.edu.tw; 3 Taiwan Space Agency, Hsinchu 300, Taiwan; cychan@tasa.org.tw (C.-Y.C.); a23919763@gmail.com (T.-Y.H.); tomlin@tasa.org.tw (C.-T.L.); 20121015jason@gmail.com (Y.-W.J.); 4 Academia Sinica, Taipei 11529, Taiwan; chihhsun.lin@phys.sinica.edu.tw

**Keywords:** attitude determination, sun sensor, trapezoidal photodiode, dual-axis fine sun sensor, Earth albedo, LEO satellites

## Abstract

This paper presents a dual-axis sun sensor employing a cross-slit aperture in conjunction with a four-quadrant trapezoidal photodiode layout. The cross-slit configuration enhances angular sensitivity and resolution, while the trapezoidal photodiode geometry preserves a high signal-to-noise ratio at both near-normal incidence and large Sun angles, maintaining reliable directional discriminability around normal incidence. Compared with conventional quad-triangle photodiode layouts, the proposed trapezoidal geometry avoids the rapid collapse of the illuminated area near the triangular apex at large incidence angles, thereby preserving signal margin near the field-of-view boundary. System-level optical verification demonstrates that, after calibration, the proposed sensor achieves an angular accuracy of ±0.3° (3σ). To mitigate performance variations induced by temperature drift, an embedded shielded dummy photodiode is incorporated to provide a dark-current reference for compensation. Unlike compensation approaches that mainly rely on pre-characterization or offline calibration, the embedded shielded dummy photodiode provides an in situ, real-time dark-current reference for compensating for temperature-induced signal drift in the actual operating environment. Experimental results under dark conditions indicate that the embedded dummy photodiode served as a dark-current reference for compensating the temperature-dependent dark-current variation in the active photodiodes, reducing the peak-to-peak dark-signal variation by 96% over a temperature range from 20 °C to 120 °C. Furthermore, a pyramid-type sun-sensor architecture is proposed by integrating the dual-axis fine sun sensor with four wide-field coarse sun sensors. This system-level configuration extends the effective Sun field of view from the conventional 120°–180° range to approximately 280°, enabling near-hemispherical Sun-angle observability for enhanced attitude determination robustness.

## 1. Introduction

**Figure 1:**
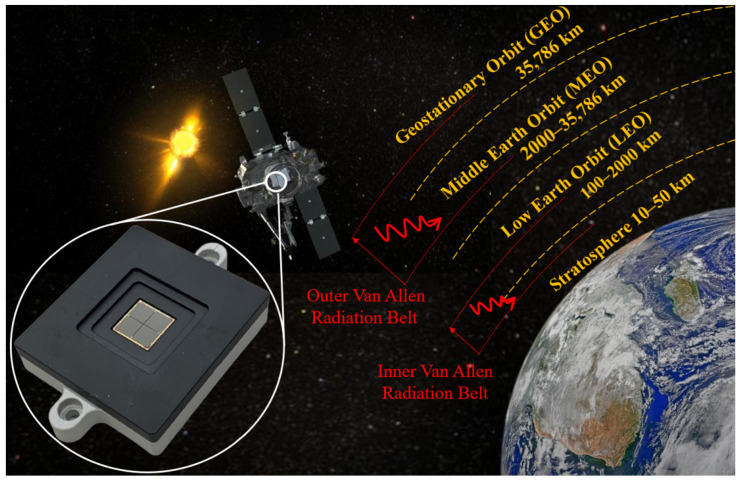
Illustration of orbital altitude classification and representative space environments, highlighting the proposed dual-axis sun sensor [16].

In recent years, Low Earth Orbit (LEO) (in Figure 1) has emerged as the most active and rapidly expanding domain of the global space industry. Driven by the proliferation of Earth observation, scientific monitoring, Internet-of-Things (IoT), and broadband communication constellations, thousands of small satellites are planned or already deployed in LEO, with projected constellation sizes reaching several tens of thousands within the next decade [1,2,3]. Compared with Medium Earth Orbit (MEO) and Geostationary Earth Orbit (GEO), LEO offers distinct advantages, including lower launch cost, reduced communication latency, higher spatial resolution for remote sensing, and greater mission flexibility. These benefits have established LEO as the primary operational regime for CubeSats and microsatellites, and have fundamentally reshaped the spacecraft system design priorities toward scalability, robustness, and cost-effectiveness in mass production.

The rapid expansion of small-satellite and CubeSat constellations in LEO has consequently imposed increasingly stringent requirements on spacecraft Attitude Determination and Control System (ADCS). In LEO missions, accurate and continuous attitude knowledge is indispensable for payload pointing, Earth observation geometry control, inter-satellite link alignment, and efficient power generation. The harsh and dynamic orbital environment characterized by high angular rates, frequent eclipse transitions, and rapidly varying Sun–Earth geometry demands an ADCS architecture that is not only precise but also robust and resilient across diverse operational phases [1,4,5]. Modern ADCS typically integrates various attitude-sensing sensors, including star trackers, inertial gyroscopes, magnetometers, and sun sensors, each contributing complementary information [3,6]. Star trackers offer the highest attitude accuracy [7], typically on the order of arcseconds, during nominal science operations; however, their performance is inherently sensitive to bright-object intrusion, high-rate slews, and temporary loss of stellar visibility during safe-mode or contingency scenarios [3,8]. Gyroscopes are subject to bias drift and noise accumulation, causing attitude errors to grow over time without external reference updates, whereas magnetometers generally provide limited accuracy because their measurements are affected by spacecraft magnetic disturbances and variations in the geomagnetic field, particularly in low-altitude orbits. Sun sensors can provide absolute Sun-vector measurements with sub-degree-level angular accuracy. Owing to the absence of complex imaging optics, sun sensors typically exhibit lower computational complexity and can be realized with a smaller size, lower mass, and reduced power consumption than star trackers [9,10,11]. In spacecraft constrained by size, weight, and power consumption (SWaP), sun sensors could be the essential attitude-sensing component where the integration of star trackers is impractical [2]. Furthermore, sun sensors can serve as a backup attitude reference during mission phases in which star trackers are unavailable or unreliable, including detumbling after launch, initial Sun acquisition, eclipse recovery, and safe-mode operations [4,12].

Modern sun sensors intended for sustained LEO deployment must achieve high angular accuracy for fine pointing and maintain uninterrupted operational availability when star trackers falter [13]. Long-term electrical stability must also be preserved under combined environmental and thermal cycling stresses corresponding to mission exposure levels [14,15]. These challenges are further exacerbated in CubeSat-class missions, where SWaP constraints limit sensor footprints to only a few square centimeters and power consumption to sub-100 mW levels [2]. Such limitations effectively preclude bulky multi-die or mechanically complex configurations. This motivates the development of compact, monolithic, dual-axis sun sensor architectures optimized for SWaP-limited platforms without compromising robustness or reliability [11].

Sun sensors are generally classified into coarse sun sensors and fine sun sensors according to their measurement accuracy. Coarse sun sensors typically provide only a single Sun azimuth estimate with a wide field of view (FOV) coverage but limited angular resolution. This functionality is commonly achieved using simple photodiode arrangements that encode the Sun direction through relative photocurrent intensity measurements [12,17,18,19]. Fine sun sensors provide two-dimensional Sun azimuth estimates with angular accuracy typically reaching the sub-degree or tenth-of-a-degree level [3,10,13]. Existing FSSs can be broadly categorized into image-based architectures [8,20,21,22] and non-imaging sensor architectures [17,23,24,25,26]. Image-based sun sensors typically consist of a sensor array combined with a lens or pinhole aperture to directly capture the solar images [8,22]. This approach provides high angular accuracy and enables flexible calibration through image processing [27,28]. However, relative to non-imaging sensor architectures, image-based sun sensors generally involve higher system complexity, increased power consumption, larger physical volume, and may be more sensitive to environmental and thermal effects [29]. In contrast, non-imaging sun sensors employ a small number of photosensitive regions, implemented either discretely or on a shared substrate, together with a geometrically shaped optical window. The incident Sun angle is then estimated from the resulting analog photocurrent distributions [23,24]. These architectures offer compact form factors, low power operation, and high robustness, making them well-suited for CubeSat and microsatellite missions [2,11]. According to prior studies, non-imaging sun sensors typically adopt square or triangular geometries for the photosensitive regions. Square configurations preserve an improved signal-to-noise ratio near the zero-angle condition and at large incident angles by maintaining a finite effective illuminated area [11,21]. However, the symmetric geometry inherently results in directional ambiguity near the origin. Triangular geometries [10,30] can enhance angular sensitivity toward the FOV boundaries; however, their effective illuminated area rapidly diminishes near the apex region, leading to signal collapse and degraded signal-to-noise ratio under large incident angles or near normal incidence.

Representative commercial sun sensors for LEO missions exhibit pronounced trade-offs among angular accuracy, update rate, physical footprint, and power consumption, as summarized in Table 1. Devices such as the Bradford Mini-FSS, Tensor Tech FSS-15M, CubeSense Sun, AAC Clyde Space SS200, and the SolarMEMS nano-SSOC series typically provide moderate to wide fields of view ranging from approximately 110° to 166°, thereby enabling basic Sun visibility during large attitude excursions. Their angular accuracy typically falls within the 0.3°–0.9° range, while long-term stability and operational lifetime are not consistently specified across products.

In addition, update rates are often relatively low (≤10 Hz) or unspecified, which may limit performance during high-rate detumbling, rapid slew maneuvers, or agile attitude transitions. This limitation is particularly relevant in LEO missions, which are characterized by rapid variations in Sun–Earth geometry, frequent eclipse transitions, and short orbital periods that demand continuous, temporally dense, and reliable Sun-vector measurements.

Furthermore, improved performance among commercially available sun sensors is often accompanied by larger physical footprints and increased mass, with representative devices exceeding 50×40×17 mm^3^ in size or weighing several hundred grams. These form factors limit mechanical placement flexibility and increase vulnerability to partial obstruction and Earth albedo contamination. In addition, most commercially available sun sensors primarily emphasize compensation for Earth’s albedo effects, whereas compensation strategies addressing angular accuracy degradation induced by temperature drift are seldom discussed or explicitly specified.

Motivated by these requirements, the sun-sensor suite, including the coarse sun sensor (CSS), the uni-axis sun sensor (UFSS), the dual-axis sun sensor (DFSS), and the panoramic pyramid-type sun sensor (PHSS), is proposed. A new compact DFSS is proposed that preserves the inherent advantages of non-imaging designs while addressing their key limitations, based on a cross-slit optical aperture combined with an asymmetric trapezoidal photosensitive geometry [16]. Compared with a square aperture, the cross slit aperture design can improve angular resolution and sensitivity by enhancing directional intensity gradients along both sensing axes, thereby strengthening the differential response to small angular variations. The asymmetric four-sensor layout ensures that the incident sunlight near the zero-angle condition produces distinguishable responses across all four photosensitive channels, thereby preserving directional information. In addition, the trapezoidal photosensitive region is adopted instead of a triangular shape to preserve a finite sensing area and maintain a robust photocurrent level. Therefore, the proposed design improves the signal-to-noise ratio (SNR), particularly at large incident angles and near normal incidence. Furthermore, an embedded dummy sensing region is implemented by shielding a dedicated photosensitive area with a metal layer to prevent optical illumination. This dummy region serves as a dark-current reference for the active photodiode, enabling real-time compensation for temperature-dependent dark-current drift. This real-time compensation scheme aims to reduce dark-current-induced offsets that may affect angular measurement accuracy during long-term orbital operation. Existing sun-sensor temperature-compensation methods mainly rely on pre-characterized temperature-dependent photoelectric models or temperature-compensation coefficients, where the measured temperature is used during operation to correct temperature-induced variations in the photodiode output [39,40]. In contrast, the proposed embedded dummy-photodiode approach does not require an additional temperature sensor. Since the dummy photodiode is fabricated on the same substrate as the active photodiodes, it experiences synchronous temperature variations and provides an in situ reference for compensating common-mode dark-current drift. Moreover, this architecture can potentially account for radiation- or aging-induced dark-current shifts, which are not directly captured by conventional temperature-sensor-based compensation methods.

A single sun sensor typically offers a FOV of about 120° to 180°, which restricts full hemispherical Sun coverage and has motivated the development of pyramid-based and panoramic sun sensor architectures [41,42,43]. For example, Cercós Pita et al. demonstrated a truncated-pyramid sun sensor composed of five coarse sun sensors, achieving an angular precision of ±1.8° after calibration and a field of view of 220° [42]. To address this limitation, this work proposes the PHSS, which extends the effective observation range to approximately 280°. The PHSS adopts a pyramid configuration in which the proposed DFSS is mounted on the top facet, while four CSS units are distributed across the inclined side facets. After calibration, both the proposed CSS and DFSS achieved sub-degree angular accuracy, suggesting that the proposed PHSS can provide sub-degree-level angle estimation. Once mounted on the satellite body, this hybrid architecture provides continuous Sun observability over a hemispherical solid angle. This configuration ensures that at least one sensing element retains a valid line of sight to the Sun under all spacecraft orientations. With the proper arrangement of two PHSSs, panoramic observation can be achieved, enabling continuous Sun observability over nearly all three-dimensional orientations and improving ADCS robustness.

Beyond optoelectronic design considerations, spaceborne sun sensors operating in LEO must function reliably under harsh orbital environments, including rapid orbital dynamics and repeated thermal excursions [1,4]. These conditions can introduce performance drift in photodiodes and readout circuits, potentially affecting long-term measurement stability [15]. Therefore, practical sun sensors must maintain stable optoelectronic behavior over wide temperature ranges to ensure reliable operation throughout the mission lifetime.

The main contributions of this paper are summarized as follows:

1. A dual-axis fine sun sensor architecture employing a cross-slit aperture and a four-quadrant trapezoidal photodiode layout is proposed [37]. The cross-slit configuration enhances angular sensitivity and resolution, while the trapezoidal geometry preserves a high SNR and reliable directional discriminability at both normal and large incidence angles. The proposed sun sensor achieved an angular accuracy of ±0.3° within three standard deviations.
2. An embedded shielded dummy photodiode is integrated as a dark-current reference to support compensation for temperature-dependent dark-current variations that may contribute to performance variations in angular sensing. Under dark measurement conditions, the proposed compensation reduces the peak-to-peak dark-signal variation by 96% over a temperature range of 20 °C to 120 °C.
3. A panoramic pyramid-type hybrid sun sensor is further proposed by integrating dual-axis fine sun sensors with four wide-field coarse sun sensors [38]. This configuration extends the effective Sun field of view to approximately 280°, enabling near-hemispherical Sun-angle observability.

To provide a comprehensive presentation of the design, implementation, and verification of the proposed sun sensor, this paper is organized as follows. Section 2 presents the sensor design considerations and the proposed architecture. Section 3 describes the fabrication process, circuit implementation, and characterization of the proposed sun sensors. Section 4 reports the system-level optical verification results. Finally, Section 5 presents the discussion and conclusions.

## 2. Sensor Design Considerations and Proposed Architecture

This section presents the key sensor design considerations and the proposed architecture.

### 2.1. Design Considerations

This subsection outlines the key design considerations that guide the proposed sun sensor architecture. The discussion focuses on trapezoidal photodiode geometry for angular sensing, an embedded dummy photodiode design for drift compensation, and an albedo detection strategy for identifying Earth-reflected illumination.

#### 2.1.1. Trapezoidal Photodiode Geometry for Robust Angle Sensing

Existing two-axis analog sun sensors generally use either four photosensitive quadrants or two orthogonally arranged single-axis sensing units. The former typically employs four symmetrically arranged and geometrically identical square photosensitive regions with a square or circular aperture [24,28,44], whereas the latter uses paired symmetrically arranged and geometrically identical triangular photosensitive regions with narrow slit apertures for axis-wise angle estimation [10,11,30]. Such geometrically identical and symmetrically arranged photosensitive regions may exhibit directional ambiguity near normal incidence, as the photocurrent signals from different sensing regions become nearly indistinguishable. At large incidence angles, the projected light spot approaches the apex of the triangular photosensitive region, causing the effective illuminated area to rapidly collapse. Consequently, the photocurrent approaches the magnitude of the intrinsic dark current, leading to severe degradation of the signal-to-noise ratio and unstable angle estimation. Although these existing sun-sensor configurations can achieve sub-degree angular accuracy through calibration, their intrinsic signal-distribution characteristics may still lead to directional ambiguity near normal incidence and degraded angle-estimation stability at large incidence angles due to signal-margin degradation. These limitations may not be fully resolved by calibration, fabrication precision, or readout-circuit improvements alone.

To overcome these limitations, a centrosymmetric trapezoidal photodiode geometry was adopted for both the UFSS and DFSS. By replacing the singular apex of a triangular layout with a finite upper base, the trapezoidal shape enforces a strictly nonzero illuminated area for all admissible incidence angles. This geometric constraint preserves sufficient signal margin even near the field-of-view boundaries. At the same time, the trapezoidal geometry maintains a well-defined lateral responsivity gradient required for high-resolution angular measurement. The corresponding geometric model and its quantitative implications on illuminated area, linearity, and sensitivity are formally derived and discussed in Section 2.2.2. The trapezoidal sensing elements are further arranged in mirror symmetry across quadrants, providing distinct but complementary sensitivity directions along the two orthogonal axes [10,12,44]. This intentional geometric diversity produces staggered peak responses among the quadrants and enables robust two-dimensional angle estimation. Importantly, this behavior reflects the designed dual-axis sensing mechanism rather than fabrication mismatch and is experimentally validated in Section 4.

#### 2.1.2. Embedded Dummy Photodiode Design for Drift Compensation

In addition to functional and geometric design considerations, spaceborne sun sensors must remain resilient to environmental disturbances. These disturbances include temperature drift, leakage current variations, and Earth albedo effects, each of which introduces signal offsets through distinct physical mechanisms [15,45]. To address these non-ideal effects, a fully metal-shielded photodiode is implemented on the same substrate as the proposed sun sensors to serve as an embedded dummy photodiode, providing a reference for compensating signal offset errors in the analog front end. By sharing identical junction characteristics while remaining optically isolated, the dummy photodiode can provide real-time measurement of dark-current variations induced by thermal conditions [27,46].

The active photodiode is the photosensitive region on the proposed sun sensor that directly receives incident illumination. The output current of the active photodiode inherently consists of photocurrent generated by incident illumination and dark current [47]. The dark current varies with temperature and long-term device variations, introducing illumination-independent drift. To mitigate this drift, the dark current of the active photodiode is estimated from the dark current measured by the dummy photodiode and subtracted from the active photodiode output. This estimation is based on a calibrated mapping function that captures the correlated dark-current behavior between the dummy and active photodiodes. The temperature-dependent characteristics of this mapping are discussed in detail in Section 3.4. Accordingly, the compensated signal can be expressed as

(1) $$I_{\mathrm{comp}}=I_{\mathrm{active}}-I_{\mathrm{dark}}\;\left(I_{\mathrm{dummy}}\right),$$

where $I_{\mathrm{comp}}$ denotes the compensated output current of the active photodiode after dark-current subtraction, and $I_{\mathrm{active}}$ represents the raw output current of the active photodiode prior to compensation. $I_{\mathrm{dark}}\left(\;\cdot \;\right)$ denotes a calibrated mapping function that predicts the dark current of the active photodiode from the dummy photodiode measurement, which inherently reflects the prevailing temperature conditions.

#### 2.1.3. Albedo Detection

Albedo refers to the diffuse solar radiation reflected from the Earth’s surface and atmosphere back toward the spacecraft. This reflected illumination introduces an additional optical signal that is independent of direct sunlight. Such additive illumination can distort the apparent irradiance distribution on the sensor and thereby bias Sun angle estimation, motivating the need for explicit albedo detection.

Since the direct solar irradiance under AM0 conditions is bounded by the solar constant [48], the net signal $S_{\mathrm{net}}$ should not exceed the theoretical solar baseline $S_{\mathrm{ref}}$. Accordingly, a signal-level albedo screening criterion could be established based on the physical upper bound of the incident flux [49,50,51]. An albedo interference flag is therefore asserted when the aggregate output surpasses this validity threshold:

(2) $$S_{\mathrm{net}}>\left(1+\delta \right)S_{\mathrm{ref}},$$

where δ represents the safety margin for orbital flux variations.

### 2.2. Proposed Architecture

Based on the design considerations outlined in Section 2.1, three types of sun sensors are proposed: CSS for wide-angle coverage, UFSS, and DFSS for high-resolution angle estimation. To achieve hemispherical field-of-view coverage without degrading angular accuracy, the four CSSs and one DFSS are further integrated into a pyramid-type hybrid sun sensor [42,43]. The geometric operating principles and associated analytical models for each configuration are presented below.

#### 2.2.1. Coarse Sun Sensor (CSS) Architecture

**Figure 2:**
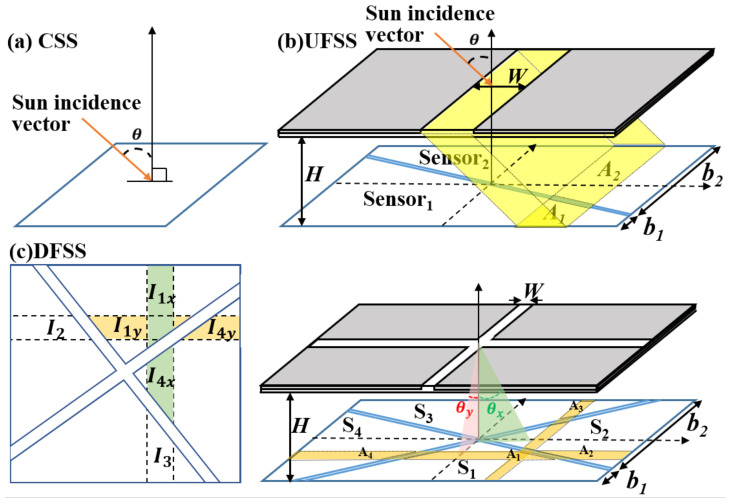
Schematic of the optical structural designs of the proposed (**a**) coarse sun sensor (CSS), (**b**) uni-axis fine sun sensor (UFSS), and (**c**) dual-axis fine sun sensor (DFSS).

The CSS can be realized with a single, simple photodiode structure, as shown in Figure 2a. When a planar photodiode is illuminated by sunlight, the generated photocurrent is proportional to the effective irradiance on the sensing surface. The irradiance with respect to the solar incidence angle follows Lambert’s cosine law [47,52]. Thus, the relationship between the ideal photocurrent and the solar incidence angle can be expressed as

(3) I=AJcosθ,

where *A* is the active area, *J* is the photocurrent density, and θ is the solar incidence angle. The angular sensitivity with respect to the photocurrent is defined as

(4) $$S_{\mathrm{CSS}}=\left|\frac{d\theta }{dI}\right|=\frac{1}{A\;J\;\sin\theta }.$$

Note that the angle θ obtained from (3) represents the angular separation between the incident Sun vector and the surface normal of the photodiode. The angular sensitivity of the CSS, as expressed in (3), exhibits a strong dependence on the incidence angle due to its cosine-law response.

In particular, near normal incidence (θ→0), the sensitivity increases sharply as sinθ approaches zero, rendering the angle estimation extremely susceptible to current fluctuations. These current fluctuations arise not only from variations in the photocurrent but also from the sensor’s dark current, leakage currents, and readout noise, thereby degrading the achievable angular accuracy. As the incidence angle approaches grazing conditions, the sensitivity decreases monotonically, resulting in a more stable but less responsive angular estimate. This characteristic makes the CSS inherently well-suited for wide-FOV Sun acquisition and coarse attitude determination, while limiting its effectiveness for fine pointing near the sensor normal. Importantly, this sensitivity profile complements that of fine sun sensors, whose differential or ratiometric architectures typically achieve higher precision at small angles but suffer from reduced observability or signal collapse near the FOV boundary. Consequently, the CSS serves as a robust wide-angle reference that bridges initial acquisition and safe-mode operation, providing reliable Sun vector information when fine sensors operate outside their optimal angular range [2, 5].

#### 2.2.2. Trapezoidal Uni-Axis Fine Sun Sensor (UFSS) Architecture

The UFSS is designed with two trapezoidal photosensitive regions that together form a rectangular sensing area, in conjunction with a rectangular transparent aperture patterned on the cover glass, as shown in Figure 2b. Sunlight entering an aperture of width *W* illuminates the photosensitive plane. The projected illumination position on the photosensitive plane shifts laterally as the solar incidence angle θ changes. *H* denotes the vertical distance between the cover glass and the photosensitive plane. Under this configuration, the angular range of solar illumination that can be received by the sensor, i.e., the FOV, can be determined as

(5) $$\mathrm{FOV}=2\tan^{-1}\;\left(\frac{W}{H}\right).$$

Sunlight incident on the two trapezoidal photosensitive regions generates two photocurrents, denoted as $I_{1}$ and $I_{2}$, respectively. The dependence of the ratiometric response, derived from these photocurrents, on the solar incidence angle can be expressed as

(6) $$R_{\mathrm{UFSS}}=\frac{I_{2}-I_{1}}{I_{2}+I_{1}}=\frac{2H(b_{2}-b_{1})}{W(b_{2}+b_{1})}\tan\theta ,$$

where $b_{1}$ and $b_{2}$ denote the upper and lower base widths of the trapezoidal photosensitive regions, respectively, and *W* is the effective aperture length. The difference between $I_{1}$ and $I_{2}$ is proportional to tanθ. By normalizing this differential signal with the sum $\left(I_{1}+I_{2}\right)$, the influence of variations in solar irradiance is inherently suppressed. Therefore, the solar incidence angle is obtained by inverting (6) as

(7) $$\theta =\arctan\;\left(\alpha R_{\mathrm{UFSS}}\right),\;\alpha =\frac{W(b_{2}+b_{1})}{2H(b_{2}-b_{1})}.$$

where the dimensionless shape factor α compactly captures the geometric dependence of the UFSS response.

The parameter α defines the fundamental design trade-off imposed by the trapezoidal geometry. A large α, corresponding to a narrow upper base width $b_{1}$, enhances the angular sensitivity and preserves the intrinsic linearity of the response. However, at large incidence angles, the effective illuminated area rapidly diminishes, leading to poor signal-to-noise ratio near the field-of-view edges and an increased risk of effective dead zones. Conversely, a small α, associated with a wide $b_{1}$, maintains a sufficiently large illuminated area over the FOV and ensures robust edge SNR. However, this comes at the cost of reduced angular sensitivity, as evidenced by a diminished current variation with respect to the incidence angle. The UFSS design space is therefore bounded by these opposing limits, and an intermediate value of α is required to simultaneously preserve angular responsiveness and maintain a non-vanishing signal margin across the FOV.

#### 2.2.3. Dual-Axis Fine Sun Sensor (DFSS) Architecture

The DFSS is a direct two-dimensional extension of the UFSS differential-area ratio formulation, enabling simultaneous estimation of the azimuth and elevation angles, $\theta_{x}$ and $\theta_{y}$. By using a single cross-shaped optical aperture at the same projection height *H* (Figure 2c), four identical photosensitive regions are arranged in a centrosymmetric quadrant configuration. As a result, each angular component is encoded by a UFSS-equivalent normalized ratio along the corresponding axis.

For the *i*-th segment (i=1,…,4), the generated photocurrent $I_{i}$ can be decomposed into its dominant orthogonal contributions,

(8) $$I_{i}=I_{ix}+I_{iy},$$

where $I_{ix}$ and $I_{iy}$ denote the principal current components associated with the projected displacements along the *x*- and *y*-axes, respectively. This decomposition is justified by the geometric independence of the orthogonal projected overlaps formed by the cross-shaped aperture.

Following the UFSS definition of the ratiometric response $R_{\mathrm{UFSS}}$, the DFSS employs axis-specific ratiometric response variables defined as

(9) $$R_{k}^{\mathrm{DFSS}}=\frac{\sum_{i\in P_{k}}I_{ik}-\sum_{j\in N_{k}}I_{jk}}{\frac{1}{2}\;I_{\mathrm{total}}},\;k\in \left\{x,y\right\},$$

where $I_{\mathrm{total}}$ is the sum of $I_{1}$, $I_{2}$, $I_{3}$, and $I_{4}$.

Here, $P_{k}$ and $N_{k}$ denote the sets of photosensitive segments contributing positively and negatively to the effective differential photocurrent along axis *k*, respectively, under a given incidence condition [44,53]. The composition of these sets depends on the illuminated regions determined by the projected spot position on the photosensitive plane, where the incidence quadrants are defined with respect to the *x*-*y* coordinate system centered at the optical axis shown in Figure 2c. For different incidence quadrants, the same formulation applies by reassigning the active segment sets according to the sign of the projected displacement along each axis.

The angular components are then reconstructed using the same functional form as the UFSS,

(10) $$\theta_{k}=\arctan\;\left(\alpha_{k}R_{k}^{\mathrm{DFSS}}\right),\;k\in \left\{x,y\right\},$$

where $\alpha_{x}$ and $\alpha_{y}$ are geometry-dependent sensitivity coefficients.

Substituting Equation (9) into Equation (10), the angular components can be explicitly expressed as

(11) $$\theta_{k}=\arctan\;\left[\alpha_{k}\frac{\sum_{i\in P_{k}}I_{ik}-\sum_{j\in N_{k}}I_{jk}}{\frac{1}{2}I_{\mathrm{total}}}\right],\;k\in \left\{x,y\right\}.$$

This explicit form directly relates the reconstructed angle to the normalized differential photocurrent.

#### 2.2.4. Panoramic Pyramid-Type Hybrid Sun Sensor (PHSS) Architecture

**Figure 3:**
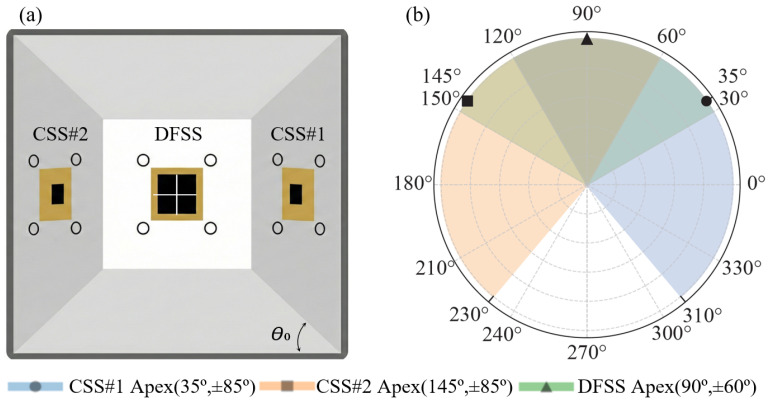
(**a**) Top view of the physical prototype of the panoramic pyramid-type hybrid sun sensor with $\theta_{0}=55{}^{\circ}$. (**b**) Polar FOV showing DFSS coverage of ±60° and CSS coverage of ±85°.

Cosine-law-based CSS provides robust wide-field Sun visibility and reliable coarse angle estimation. However, its sensitivity characteristics inherently limit the achievable accuracy near normal incidence, where dark current and readout noise dominate the angular error budget. Conversely, fine sun sensors exhibit superior angular resolution in small-angle regimes but typically suffer from degraded SNR at large incidence angles. This complementary behavior motivates a hybrid sensing strategy that integrates both coarse and fine sensing principles within a unified architecture. Thus, a PHSS is proposed to exploit this complementarity by combining multiple wide-FOV CSS elements with fine-resolution sensing surfaces arranged in a three-dimensional pyramidal geometry. A DFSS is placed at the apex, while four CSS units are mounted on the inclined facets (Figure 3). This configuration enables continuous Sun vector observability over a panoramic angular range exceeding that of a single planar sensor, while simultaneously preserving high angular precision within the central FOV (>2π). The pyramid topology ensures that at least one sensing facet operates within its optimal sensitivity region across virtually all Sun incidence angles, thereby avoiding both the SNR regime of fine sensors near FOV boundaries and the noise-dominated regime of CSS near normal incidence. By fusing the coarse and fine measurements in a geometry-aware manner, the hybrid sensor achieves a balanced performance envelope that unifies large angular coverage, high estimation fidelity, and operational robustness. This architecture is particularly advantageous for LEO missions involving large-angle maneuvers, eclipse recovery, and rapid attitude transitions, where uninterrupted availability of the Sun vector and high accuracy are required simultaneously. The PHSS therefore represents a system-level solution that transcends the inherent limitations of individual CSS or fine-sensor designs, enabling wide-FOV acquisition and high-precision attitude determination in a compact, SWaP-efficient implementation.

For a facet inclination angle $\theta_{0}$, the photocurrents of an opposing CSS pair under an incident angle θ are modeled as

(12) $$I_{k}\left(\theta \right)=I\left(0\right)\cos\;\left(\theta_{0}+{\left(-1\right)}^{k}\theta \right),\;k=1,2.$$

where I(0) denotes the photocurrent at normal incidence to each facet. $I_{1}$ and $I_{2}$ represent the photocurrents generated by CSS1 and CSS2, respectively. Taking the differential signal yields

(13) $$\Delta I=I_{1}-I_{2}=2I\left(0\right)\sin\theta_{0}\sin\theta .$$

Letting $C=2I\left(0\right)\sin\theta_{0}$, the incident angle is written as

(14) $$\theta =\arcsin\;\left(\frac{\Delta I}{C}\right).$$

The two opposing CSS units are tilted by ±35° with respect to the surface normal, corresponding to ±55° relative to the substrate plane, each providing a local FOV of ±85°. Consequently, the hybrid configuration enables an effective panoramic field of view of approximately 280° when combined with the central ±60° coverage of the DFSS. This differential formulation, obtained by subtracting the outputs of the two opposing CSS elements, effectively converts the two strongly nonlinear $\cos(\theta_{0}\;\pm \;\theta )$ responses into an approximately linear sinθ term. As a result, nonlinearity near grazing incidence is mitigated, enabling continuous, hemisphere-wide Sun acquisition without mechanical scanning, as validated experimentally in Section 4 [4].

## 3. Fabrication, Circuit Implementation, and Characterization

This section describes the realization and validation of the proposed sun-sensor suite, spanning device fabrication, packaging integration, readout circuit design, and electrical and optoelectronic characteristics of the proposed sun sensor.

### 3.1. Fabrication and Packaging

**Figure 4:**
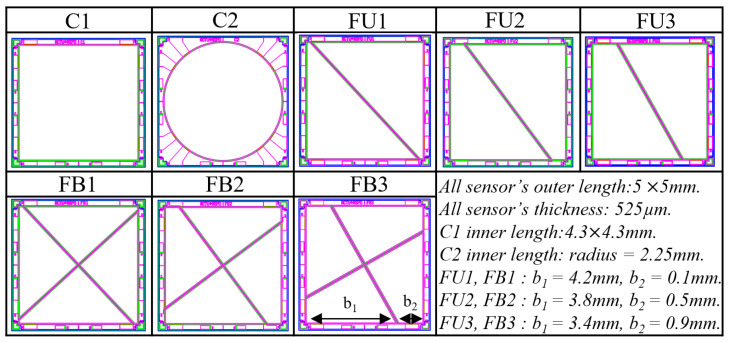
Geometric specifications and layouts of the proposed sun sensor variants.

To evaluate the trade-offs among angular accuracy, linearity, and field of view, eight sensor variants were investigated across three types of sun sensors. These include the CSS series with C1 and C2, the UFSS series with FU1, FU2, and FU3, and the DFSS series with FB1, FB2, and FB3. Within the FU and FB series, key geometric parameters were parametrically swept to quantify their influence on the linearity factor α and the resulting angular sensitivity. The geometric specifications and layout topologies of all variants are summarized in Figure 4.

**Figure 5:**
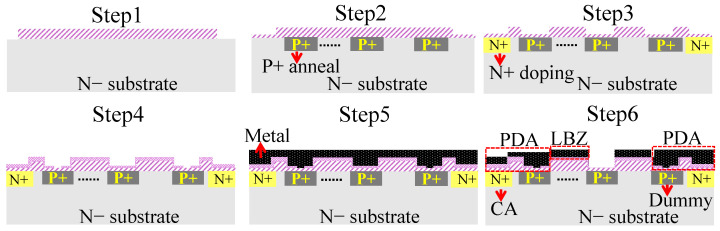
CMOS-compatible fabrication process flow (Steps 1–6).

The sun sensor prototypes were fabricated on 6-inch N-type silicon wafers using a standard CMOS-compatible process to ensure reproducibility and device stability. The fabrication sequence, depicted in Figure 5, starts with N^−^ epitaxial growth (Step 1), followed by patterned P^+^ diffusion to realize the trapezoidal photodiode geometry (Step 2) and localized N^+^ diffusion to form low-resistance ohmic contacts (Step 3). After passivation deposition and etching (Step 4), a metal layer is deposited and patterned (Steps 5–6) to define electrical interconnects, contact apertures (CA), bonding pads (PAD), and metal-shielded dummy structures adjacent to the active photodiodes. These dummy devices, each with a physical dimension of 1100μm×170μm, share identical process and junction characteristics with the active photodiodes and provide on-chip references for temperature-dependent current compensation. A final post-deposition anneal reduces implantation-induced defects, lowers dark current, and improves long-term junction stability.

**Figure 6:**
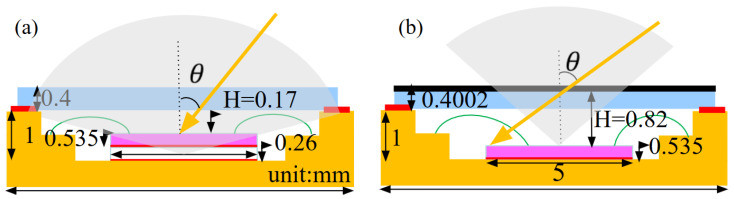
Cross-sectional packaging geometry of the (**a**) CSS and (**b**) FSS. Black: Cr aperture layer; Blue: quartz cover glass with AR coating; Pink: silicon die; Red: die-attach adhesive; Orange: LCC-48 ceramic package body.

The operational mode and target FOV are primarily determined by the optical height (*H*) defined within the LCC-48 ceramic carrier. As illustrated in Figure 6, the packaging stack-up distinguishes the CSS from the FSS. For the FSS configuration, a silicon spacer maintains an optical height of H≈0.82 mm, yielding an effective FOV of approximately ±60°. In contrast, the CSS employs a reduced baffle height of H≈0.17 mm to minimize shadowing and enable near-hemispherical coverage up to ±85°.

The die is bonded to the LCC-48 ceramic carrier using an adhesive layer of approximately 50 μm. A quartz cover glass (${\mathrm{SiO}}_{2}$) with an anti-reflection coating provides high optical transmittance (∼98%). A 200 nm chromium (Cr) layer deposited on the cover glass serves as the optical shield and is patterned to form the slit aperture in the configuration. Electrical interconnection and optical alignment follow standard LCC-48 packaging practices and do not limit the measured angular accuracy.

### 3.2. Electrical Characterization

**Figure 7:**
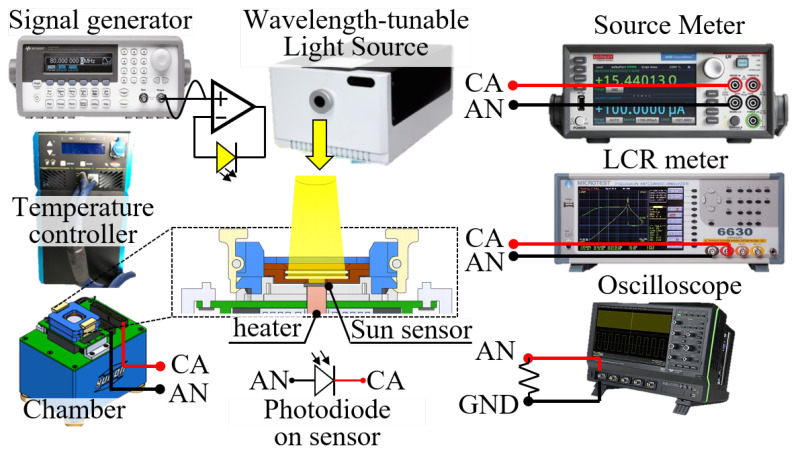
Experimental setup for the electrical characterization of the proposed sun sensors.

Device-level electrical characterization was performed using a Keithley 2450 SourceMeter for current-voltage (I-V) measurements and a Microtest 6630 LCR meter for capacitance-voltage (C-V) analysis. The experimental setup is illustrated in Figure 7. To minimize ambient optical interference, measurements were conducted within a shielded dark enclosure. The structural and electrical parameters for the rectangular C series, square FU series, and trapezoidal FB series configurations are listed in Table 2.

The junction capacitance ($C_{j}$) confirms the bandwidth advantage of the proposed design. As listed in Table 2, the trapezoidal FB variant exhibited a capacitance of 320 pF at −5 V, which was approximately 75% lower than the 1310 pF measured for the rectangular C series variant. This significant reduction validated the area-saving layout shown in Figure 4 and directly contributed to the enhanced high-speed response.

Regarding reliability, the breakdown voltage ($V_{BR}$), defined at 100 μA, exceeded 51.0 V for the FB series sensor, providing sufficient headroom to avoid premature breakdown. The extracted $V_{BR}$ showed a positive temperature coefficient of 43 mV °C^−1^, consistent with an avalanche breakdown mechanism that inherently mitigates the risk of thermal runaway.

**Figure 8:**
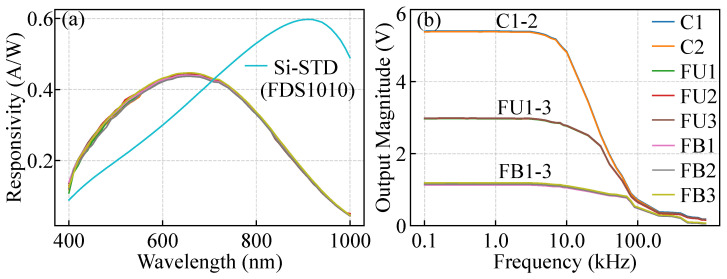
Optoelectronic performance of the proposed photodiodes. (**a**) Measured EQE versus wavelength. (**b**) Normalized frequency response of C1, FU1, and FB1.

The optoelectronic performance was evaluated to verify the sensor response under controlled optical illumination. The external quantum efficiency (EQE) was derived from spectral responsivity measurements using a wavelength-tunable light source. The measured spectral responses of the proposed sun sensors are shown in Figure 8a. The response values were referenced to a Si-STD photodiode (FDS1010), serving as a primary standard calibrated by a national metrology institute. This spectral coverage overlaps well with the high-irradiance region of the AM0 solar spectrum, ensuring efficient photon collection for sun-sensing applications.

The normalized frequency response indicates that the larger photosensitive area of the C-series variant is limited to a −3 dB bandwidth of 17 kHz, primarily due to its higher junction capacitance, as shown in Figure 8b. In contrast, the proposed FB1 variant achieved a significantly wider bandwidth of 70 kHz. This fourfold improvement in bandwidth is inversely proportional to the reduction in junction capacitance, which decreases from 1310 pF to 320 pF, as listed in Table 2.

### 3.3. Readout Circuit Implementation

**Figure 9:**
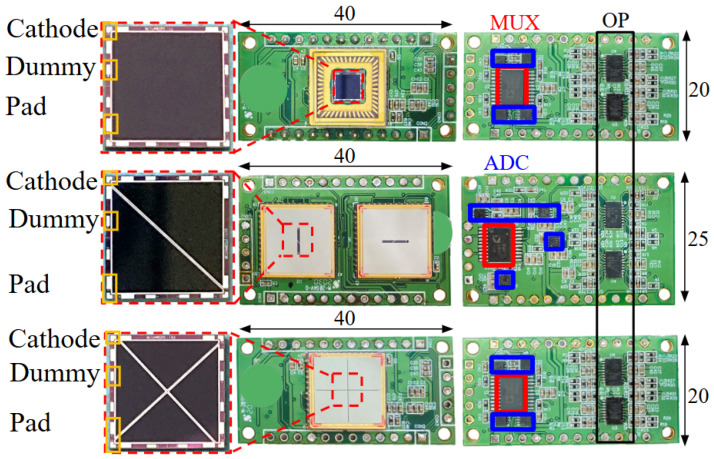
Physical implementation of the proposed coarse sun sensor, uni-axis fine sun sensor, and dual-axis fine sun sensors.

A discrete readout circuit was developed to validate the proposed sun-sensor suite at the system level. The PCB implementation integrates the packaged sensor and the discrete analog-to-digital readout circuits, as shown in Figure 9.

**Figure 10:**
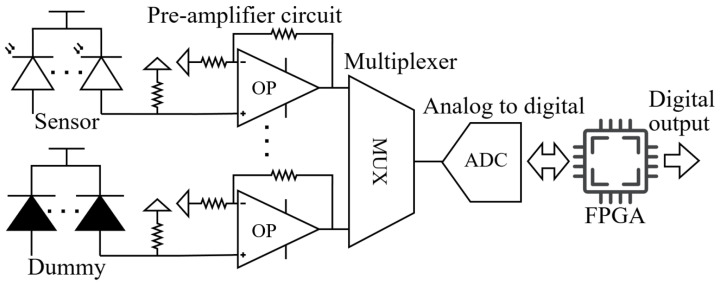
Block diagram of the sun-sensor readout circuit.

Both the active photodiode and the embedded dummy photodiode are interfaced with identical transimpedance readout circuits, in which the photocurrent is converted into a voltage signal through a parallel-connected feedback resistor and a forward amplifier. As shown in Figure 10, this shared readout configuration enables consistent signal conditioning for both sensing and reference channels. The resulting voltage outputs from either the active or dummy photodiode are selected via an analog multiplexer. The selected signal is subsequently digitized by an analog-to-digital converter (ADC) and delivered to the Field-Programmable Gate Array (FPGA) for signal acquisition and processing. The dummy photodiode output provides a reference for tracking dark current variations and improving output stability.

**Figure 11:**
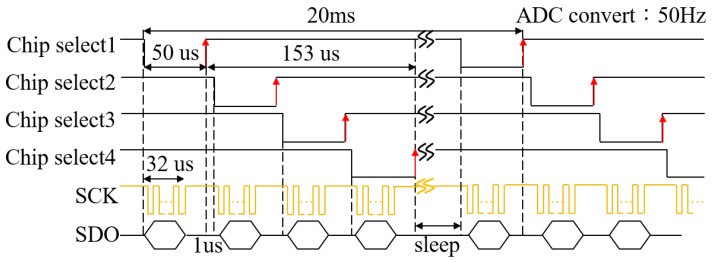
Timing diagram of FPGA control signals configured for a 50 Hz sampling rate.

Taking the DFSS signal readout as an example, four ADCs operate in a quasi-parallel manner to digitize the sensor outputs from the four active photodiodes. The FPGA retrieves the ADC data via a Serial Peripheral Interface (SPI), and the corresponding readout timing diagram is illustrated in Figure 11. Under the current implementation, the sensor signals can be sampled at a rate of up to 50 Hz. This sampling capability enables the subsequent angle estimation to be performed at an update rate of 50 Hz.

### 3.4. Thermal Drift Compensation

As described in Section 2.1.2, the proposed compensation scheme aims to estimate the dark-current variation in the active photodiode using the dark current measured by the dummy photodiode. Therefore, temperature characterization experiments were conducted under dark conditions to establish the relationship between temperature and the dark currents of both the active and dummy photodiodes.

The experimental setup is shown in Figure 7. The packaged sensor is placed inside a custom-designed thermal chamber, where the chip temperature is directly controlled through a metallic thermal contact from the bottom of the chamber. The top of the chamber is equipped with a double-layer glass window, allowing the sensor to be either illuminated or kept in darkness. In this experiment, the optical path was fully blocked since only dark current measurements were performed. The photodiode current is conducted through the package leads via metallic contacts at the chamber base and measured using a SourceMeter.

**Figure 12:**
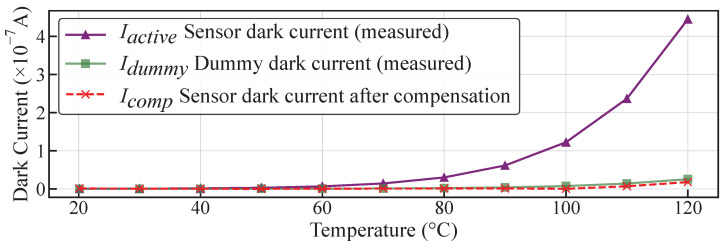
Comparison of measured and compensated currents versus temperature.

The dark currents of the active and dummy photodiodes over the temperature range of 20 °C to 120 °C are shown in Figure 12. Both currents increased exponentially with temperature, consistent with the theoretical Arrhenius relationship [47]:

(15) $$I\propto T^{2.54}e^{-E_{g}/kT}.$$

According to this formulation, the photodiode current becomes linear with temperature after logarithmic transformation. Therefore, the measured active and dummy dark currents, denoted as $I_{\mathrm{active}}$ and $I_{\mathrm{dummy}}$, are converted to the logarithmic domain. In this domain, the relationship between the two currents can be expressed as

(16) $$\log_{10}\;\left(\frac{I_{\mathrm{active}}}{I_{0}}\right)=a\;\log_{10}\;\left(\frac{I_{\mathrm{dummy}}}{I_{0}}\right)+b,$$

where $I_{0}=1\;\mathrm{nA}$ is a reference current introduced to normalize the logarithmic arguments, and *a* and *b* are fitting coefficients obtained from linear regression of the temperature characterization data. Accordingly, the temperature-dependent dark current of the active photodiode can be predicted from the dummy photodiode current as

(17) $$I_{\mathrm{pred}}=I_{0}\;\cdot \;10^{a\log_{10}\left(I_{\mathrm{dummy}}/I_{0}\right)+b}$$

The temperature-induced current drift of the active photodiode can then be compensated by subtracting this predicted component:

(18) $$I_{\mathrm{comp}}=I_{\mathrm{active}}-I_{0}\;\cdot \;10^{a\log_{10}\left(I_{\mathrm{dummy}}/I_{0}\right)+b}$$

where $I_{\mathrm{comp}}$ denotes the compensated current. The compensation parameters are fixed to a=1.07 and b=1.81 based on logarithmic fitting of the temperature characterization data. Since the dark current is generally proportional to the photodiode junction area under the same process, bias, and temperature conditions, the fitted scaling factor *b* can be compared with the effective area ratio between the active and dummy photodiodes. As described in Section 3.1, the effective areas of the active and dummy photodiodes are approximately $12.5\;{\mathrm{mm}}^{2}$ and $0.187\;{\mathrm{mm}}^{2}$, respectively, yielding an effective area ratio of 12.5/0.187≈66.8. This value is reasonably close to the corresponding scaling factor $10^{1.81}\approx 64.6$.

As shown in Figure 12, under dark conditions, the proposed compensation suppresses temperature-induced dark-current drift. A pronounced reduction in dark-current variation is observed at temperatures above approximately 70 °C.

## 4. System-Level Optical Verification

This section presents the system-level optical verification of the proposed sun-sensor suite. The laboratory optical test was used to characterize and quantitatively compare the geometric angular response and post-calibration angular residuals of the CSS, UFSS, and DFSS under controlled and repeatable illumination conditions. In addition, the capability to detect albedo-induced interference is investigated.

### 4.1. Optical Verification Setup

**Figure 13:**
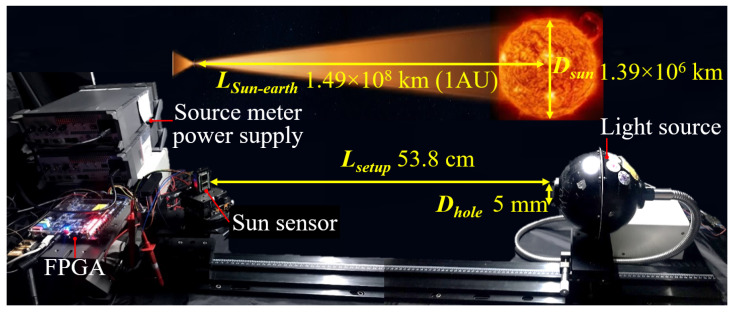
High-precision angular measurement system. **Top**: Solar angular subtense model. **Bottom**: Experimental setup for optical verification.

System-level characterization, including linearity, angular accuracy, and FOV verification, was performed using an automated optical testbed housed in a light-tight enclosure, as shown in Figure 13. A broadband halogen light source, the Illumination Technologies 3900e (Illumination Technologies, Inc., Elbridge, NY, USA), coupled with an integrating sphere was used to generate a highly uniform Lambertian beam with spatial non-uniformity below 1%. The emitted spectrum spans 400 nm to 1000 nm, which fully covers the peak responsivity range of the proposed sun sensors.

To replicate the apparent solar angular diameter observed from LEO of approximately 0.53°, the integrating-sphere aperture diameter $D_{\mathrm{hole}}$ was set to 5 mm. The source-to-sensor distance $L_{\mathrm{setup}}$ was set to 53.8 cm. This geometry yields an effective angular subtense of $\theta \approx \arctan\left(D_{\mathrm{hole}}/L_{\mathrm{setup}}\right)\approx 0.53{}^{\circ}$, ensuring a quasi-parallel illumination geometry representative of solar incidence [11,41]. The incident irradiance was calibrated to approximately 5 × 10^−2^ W m^−2^ using a NIST-traceable reference photodiode. This irradiance level was selected to provide measurable photodiode signals under the laboratory test conditions. The optical setup is primarily intended for geometric and functional validation rather than absolute solar-irradiance emulation.

The sun sensor response was characterized under varying incident illumination angles. The incident angle was systematically varied using a two-axis optical rotation stage with a resolution of 0.01°. The rotation axis was aligned with the geometric center of the sun sensor to minimize parallax-induced errors. Signals from all active and dummy photodiodes were synchronously digitized and acquired by the FPGA. The acquired data were subsequently processed using the polynomial calibration algorithms presented in Section 2.

### 4.2. Angular Accuracy and Linearity

#### 4.2.1. Coarse Sun Sensor (CSS)

**Figure 14:**
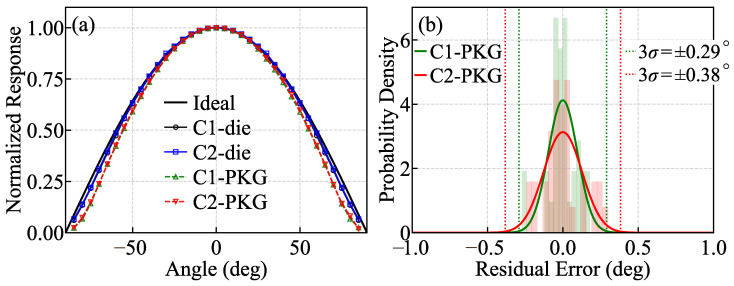
Optical verification of the proposed coarse sun sensors. (**a**) Normalized angular responses. (**b**) Post-calibration angular error of the packaged sensors.

The CSS had two photosensitive layout designs, including a square layout C1 and a circular layout C2. The angular responses of C1 and C2 were measured at both the die and packaged levels. The measured normalized angular responses are compared with the ideal cosine model in Figure 14a. The response of C2 is closer to the ideal cosθ characteristic [11,12], whereas C1 showed slightly larger deviations due to edge-related nonuniformity in the effective photosensitive area. To correct geometry- and packaging-induced nonlinearities, polynomial calibration was applied to the measured angular responses, where the higher-order polynomial terms are physically motivated by the nonlinear cosine-type angular response over the wide field-of-view operating range. The residual angular errors after ninth-order calibration are presented for the CSS variants in Figure 14b. The residual angular error is defined as the difference between the ninth-order calibrated angle and the reference incident angle at each sampled position. The 3σ angular errors, defined as three times the standard deviation of the residual error distribution, were ±0.29° for C1 and ±0.38° for C2 in the packaged configuration.

#### 4.2.2. Uni-Axis Fine Sun Sensor (UFSS)

**Figure 15:**
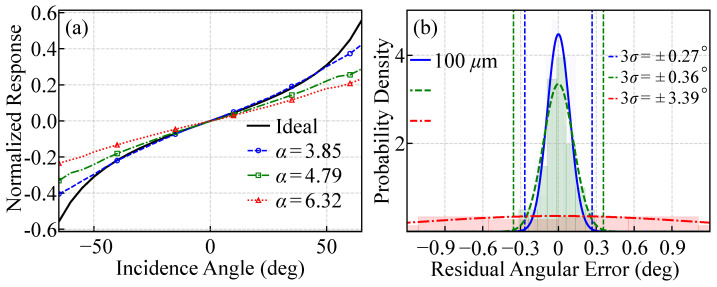
Comparison of the proposed uni-axis fine sun sensor with different α values and aperture widths. (**a**) Ratiometric responses for α={3.85,4.79,6.32}. (**b**) Effect of aperture width (W={100,500,1000}μm) for α=3.85.

The UFSS incorporated three photosensitive layout designs, with corresponding shape factors of α=3.85, 4.79, and 6.32 for FU1, FU2, and FU3, respectively, as shown in Figure 4. These designs were fabricated to investigate the impact of the shape factor on the intrinsic linearity of the ratiometric response $R_{\mathrm{UFSS}}$ with respect to the solar incidence angle. The measured ratiometric responses of these designs are compared with the ideal geometric response in Equation (6), as illustrated in Figure 15a. The ideal geometric response corresponds to a conventional triangular photodiode layout and serves as the reference for comparison with the proposed trapezoidal geometries. The α=3.85 geometry showed the closest agreement with the ideal response over the full angular range, indicating the highest intrinsic linearity. This behavior is consistent with the design principle that linearity improves as the trapezoidal geometry approaches the ideal triangular responsivity profile, yet a finite upper base is preserved to prevent apex-induced illuminated-area collapse observed in conventional triangular layouts.

The influence of the transparent aperture width *W* was further evaluated for α=3.85 using aperture widths of 100 μm, 500 μm, and 1000 μm. The residual angular error distributions after 11th-order polynomial calibration are shown in Figure 15b. The 100 μm aperture width yielded the smallest 3σ angular errors of ±0.27°, whereas larger aperture widths exhibited progressively degraded accuracy. Based on these results, DFSS verification was conducted using a cover glass with a transparent aperture width of 100 μm and the FB1 design with α=3.85 to evaluate angular accuracy.

#### 4.2.3. Dual-Axis Fine Sun Sensor (DFSS)

**Figure 16:**
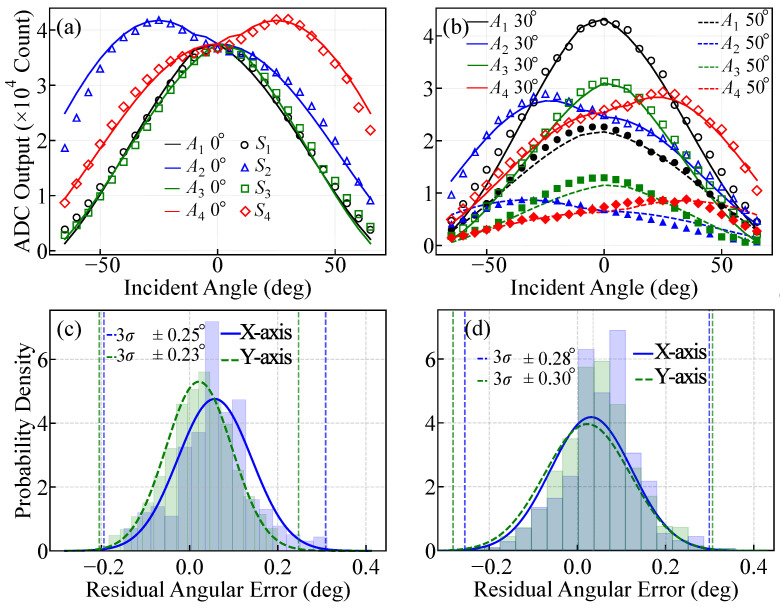
System-level optical verification of the proposed dual-axis fine sun sensor. (**a**,**b**) Measured sensor response *S* and ideal geometric response *A* with $\theta_{x}$ swept over [−60°,60°] at fixed $\theta_{y}=0{}^{\circ}$, 30°, and 50°. (**c**) Residual error after ninth-order calibration for two-axis operation. (**d**) Residual error obtained from an independent random-point validation experiment using the fixed ninth-order calibration coefficients derived in (**c**).

The measured photocurrent responses of the DFSS are shown in Figure 16a,b, where Figure 16a corresponds to $\theta_{y}=0{}^{\circ}$, while Figure 16b includes the cases of $\theta_{y}=30{}^{\circ}$ and 50°, with $\theta_{x}$ swept over the range of [−60°,60°]. The curves labeled $S_{1}$ to $S_{4}$ represent the corresponding photocurrent responses of the photosensitive regions $S_{1}$ to $S_{4}$ illustrated in Figure 2c. The curves $A_{1}$ to $A_{4}$ represent the ideal projected area-weighted photocurrents corresponding to $S_{1}$ to $S_{4}$, respectively, derived under the same incidence angles as those used in the UFSS experiments.

The peak responses of individual photodiodes occurred at different $\theta_{x}$ positions, reflecting their distinct principal sensitivity directions imposed by the trapezoidal photosensitive geometry. If all photodiodes exhibited coincident peak responses at the same angular location, their local sensitivities dI/dθ would simultaneously approach zero. This would result in a loss of angular discriminability and lead to an ill-conditioned reconstruction problem in that region. In contrast, the peak locations in A and S are staggered across the angular domain. When one photodiode enters a locally flat response region, other photodiodes retain finite sensitivity, preserving angular resolution over the entire FOV. The distributions of the calibrated two-axis residual angular errors are shown in Figure 16c. The experimental 3σ angular accuracies of the DFSS were ±0.25° along the x-axis and ±0.23° along the y-axis. To further validate the calibrated performance, an additional random-point validation experiment was conducted by measuring 1000 randomly selected angular positions in both the X and Y directions. The validation results shown in Figure 16d indicate that the 3σ angular errors remained within 0.30° for both axes, indicating that the overall measurement residuals were maintained below 0.1° level under the present experimental conditions, while also mitigating the concern of overfitting to the original calibration dataset.

#### 4.2.4. Panoramic Pyramid-Type Hybrid Sun Sensor (PHSS)

**Figure 17:**
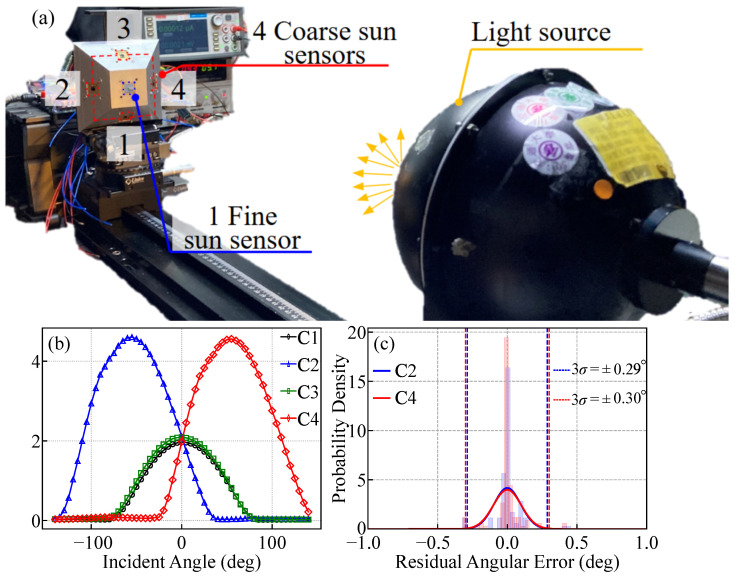
Experimental verification of the proposed panoramic pyramid-type sun sensor. (**a**) Test setup. (**b**) Differential angular response versus ideal model. (**c**) Calibrated angular error over ±150°.

To validate the proposed PHSS concept, a pyramid-type hybrid module with an inclination angle of $\theta_{0}=55{}^{\circ}$ was fabricated. This inclination angle was chosen to ensure sufficient FOV overlap between the top-mounted DFSS and the side-mounted CSS within a compact structure. This experiment primarily focused on verifying the angular response and error behavior of the side-mounted CSS. The same integrating-sphere illumination setup and rotation stage described in Section 4.1 of Section 4 was used in the experiment. During the experiment, the module was rotated about the *X*-axis, and the photocurrents from the four side-mounted CSS were recorded, as illustrated in Figure 17a. The normalized angular responses of the four CSS are illustrated in Figure 17b. CSS sensors C2 and C4 exhibited a double-peaked angular response and therefore dominated the differential signal along the *X*-axis. Similarly, the angular component along the orthogonal direction can be resolved by CSS sensors C1 and C3. The calibrated residual angular error distributions are depicted in Figure 17c, with both axes achieving 3σ angular errors within ±0.3°. This result could support the sub-degree accuracy of the pyramid-type hybrid configuration.

### 4.3. Albedo Interference Detection and Validation

The detection of Earth albedo interference follows the irradiance-bounded criterion introduced in Section 2.1.3. The net total irradiance $S_{\mathrm{net}}$ was obtained by summing the photocurrents from all photodiodes in the sun sensor and subtracting the corresponding dark current. The albedo decision is determined by the magnitude of the external flux. Under direct solar illumination, the aggregate signal is physically bounded by the solar constant. Any significant exceedance beyond this limit indicates the presence of an additional diffuse light source. Accordingly, an albedo condition is declared when $S_{\mathrm{net}}>\left(1+\delta \right)S_{\mathrm{ref}}$, where $S_{\mathrm{ref}}$ denotes the nominal solar baseline and δ=0.10 provides a safety margin for orbital irradiance variations. This criterion is applied here to experimentally validate the proposed albedo detection strategy.

As summarized in Table 3, the albedo-like secondary illumination increases the aggregate photocurrent to 15,460 counts. After dark-current offset removal, the resulting net signal is 14,961 counts, which remains above the validity threshold of 13,088. A concurrent increase is observed across all four photodiodes, with increments ranging from +691 to +1122 counts. This behavior exhibits a common-mode increase in external illumination induced by an albedo-like component. Under this signal-level laboratory test condition, the proposed irradiance-bounded criterion can identify such an increase while reducing ambiguity from internal electrical drift or photocurrent response mismatch.

## 5. Conclusions and Discussion

This work presents a compact and scalable sun-sensor suite for LEO missions, comprising a CSS, a UFSS, and a DFSS, all implemented using a CMOS-compatible process. By integrating a cross-slit aperture with optimized asymmetric trapezoidal photodiode geometries, high angular performance is achieved. This non-imaging, slit-based sun-sensor architecture eliminates bulky optical components and enables the photodiode array to be implemented within a limited silicon area, making the sensors well-suited for SWaP-constrained satellite platforms.

Geometric optimization was conducted to explore the impact of photodiode geometry and slit width on the angular response of the proposed sun sensors. In the CSS designs, two photosensitive geometries were examined to evaluate their effects on cosine-response conformity and angular accuracy. The experimental residual angular errors were approximately ±0.29°. These angular errors may be influenced by refraction and angle-dependent transmission through the cover glass.

For the UFSS designs, the experimental results indicate that a shape factor of α=3.85 yields the maximum response slope while closely approximating the ideal tangent behavior. This linearity arises as the photosensitive geometry approaches an ideal triangular responsivity profile. However, a purely triangular geometry suffers from a rapid reduction in illuminated area near the apex at large incidence angles, leading to severe signal attenuation. Therefore, the trapezoidal geometry represents a practical compromise by retaining a finite upper base, albeit at the expense of a modest reduction in linearity. In addition, a cover glass with an aperture width of W=100μm minimizes angular errors, which may be attributed to a reduced illumination footprint and lower sensitivity to stray light. As a result, the UFSS with α=3.85 and W=100μm achieves a 3σ angular accuracy of ±0.27°.

The DFSS further realizes dual-axis sensing by monolithically integrating four trapezoidal photosensitive regions on a single die, reducing the active sensing area by approximately 50% compared with two discrete uni-axis sensors while maintaining comparable angular performance. Experimental results demonstrate that the DFSS achieves angular accuracies ±0.3°.

The PHSS architecture, combining a central DFSS with four surrounding CSS units, extends the effective Sun-vector observability to a FOV of approximately 280°. This wide-coverage sensing capability enables seamless transition between coarse Sun acquisition and fine pointing, providing a unified sensing solution for detumbling, eclipse recovery, and nominal attitude control in LEO missions. Experimental results further demonstrate that an angular accuracy of approximately ±0.3° at the 3σ level can be maintained across this extended FOV.

To enhance long-term stability under varying environmental conditions, a dedicated dummy sensing region is incorporated on the same die as the active sensing region. By shielding this region with a metal layer, optical illumination is effectively blocked, allowing it to function as an on-chip reference for dark current monitoring. This reference has the potential to support real-time compensation of temperature-dependent dark-current drift, which may help reduce dark-current-induced offsets affecting angular measurements during extended orbital operation. Under dark measurement conditions, the proposed compensation strategy reduced the peak-to-peak dark-signal variation by 96% over a temperature range from 20 °C to 120 °C. Although the experimental validation in this work is limited to this temperature range, the observed compensation behavior indicates that the proposed approach can effectively suppress temperature-induced drift within this range. Based on this trend, the same method is expected to remain effective over the wider operating temperature range typically required for spaceborne sensors, from −40 °C to 120 °C.

This paper focuses on the architectural design and system-level realization of a compact sun-sensor suite, including geometric optimization, readout circuit design, and comprehensive optical verification. The experimental results validate the effectiveness of the proposed sensor architectures and compensation strategies under tested laboratory conditions, establishing a foundation for potential spaceborne operation in LEO missions.

## 6. Patents

The work presented in this study is related to the following patents:

Ou-Yang, M.; Yan, Y.-J.; Huang, G.-Y.; Cheng, T.-Y.; Liu, C.-H.; Liu, Y.-S.; Jan, Y.-W.; Chan, C.-Y.; Hsieh, T.-Y. Device and method for detecting a light irradiating angle. U.S. Patent 12,216,001, 2025.

Ou-Yang, M.; Yan, Y.-J.; Cheng, T.-Y.; Huang, G.-Y.; Liu, C.-H.; Liu, Y.-S.; Liu, Y.-W.; Chan, C.-Y.; Hsieh, T.-Y. Polyhedron device for sensing light rays. U.S. Patent 11,777,442, 2023.

## Figures and Tables

**Table 1:** Comparison of Representative Commercial Sun Sensors and the Proposed Sun Sensors.

Model	Type	FOV	Accuracy	Rate	Size	Mass	Power	Output	Life	Albedo	Comp. ^‡^
		(deg)	(3σ **^†^)**	(Hz)	(mm^3^)	(g)	(mW)		(yrs)		
Bradford CSS [31]	CSS	±90	0.4°	N/A	110 × 110 × 30	215	N/A	Ana.	N/A	N/A	N/A
AAC Clyde Space SS200 [32]	CSS	±55	0.9°	10	35 × 24 × 15	350	40	Dig.	N/A	Yes	N/A
Bradford Mini-FSS [33]	DFSS	±64	0.4°	N/A	50 × 46 × 17	50	N/A	Ana.	N/A	N/A	N/A
Tensor Tech FSS-15M [34]	DFSS	±60	0.6°	8	22 × 15 × 5.3	3	56.3	Dig.	N/A	Yes	N/A
CubeSense Sun [35]	DFSS	±83	0.3°	2	35 × 22 × 24	14.2	174	Dig.	≥5	Yes	N/A
SolarMEMS nano-SSOC [36]	DFSS	±60	0.5°	50	43 × 14 × 5.9	6.2	75.9	Dig.	N/A	Yes	N/A
**CSS of this work**	**CSS**	**±85**	**0.29°**	**N/A**	**40 × 20 × 10**	**6**	**N/A**	**Dig./Ana.**	≥5	**No**	**Yes**
**UFSS of this work**	**UFSS**	**±60**	**0.27°**	**50**	**40 × 25 × 10**	**8**	**40**	**Dig./Ana.**	≥5	**Yes**	**Yes**
**DFSS of this work [37]**	**DFSS**	**±60**	**0.30°**	**50**	**84 × 54 × 21** ^‖^	**83.9**	**40**	**Dig./Ana.**	≥5	**Yes**	**Yes**
**PHSS of this work [38]**	**Hybrid**	**±140**	**0.23–0.30°** ^§^	**50**	**65 × 65 × 35**	**<50**	**200**	**Dig./Ana.**	≥5	**Yes**	**Yes**

^†^ Accuracy values are reported as specified by manufacturers or literature; definitions (e.g., 3σ, RMSE, or maximum error) may differ. ^‡^ The compensation includes temperature-induced drift. ^§^ The reported accuracies include the DFSS-dominated central region and the CSS-dominated outer facets. ^‖^ The reported size and mass of the proposed CSS and UFSS exclude the housing, whereas those of the DFSS include the housing.

**Table 2:** Electrical parameters and temperature dependence of the proposed sensors.

Parameter	C/FU/FB @ 25 °C	Temp. Coefficient
$V_{f}$ @ 1 mA	0.72/0.70/0.68 V	−1.6 mV °C^−1^
$V_{\mathrm{BR}}$ @ −100 μA	47.5/49.0/51.0 V	43 mV °C^−1^
$R_{s}$	3.28/3.42/3.30 Ω	<5% (stable)
$C_{j}$ @ 10 kHz	1310/680/320 pF	<5% (stable)

**Table 3:** Simulation of albedo interference detection.

Parameter	Sensor 1	Sensor 2	Sensor 3	Sensor 4	Sum
Dummy Dark ($D_{\mathrm{dummy}}$)	16	17	14	16	63
Sensor Dark ($D_{\mathrm{sensor}}$)	124	135	112	128	499
Nominal ($S_{\mathrm{ref}}$)	2879	3713	2358	2949	11,899
With Albedo ($S_{\mathrm{raw}}$)	3767	4835	3049	3809	15,460
Increment (Δ)	+888	+1122	+691	+860	+3561

## Data Availability

The data presented in this study are available on request from the corresponding author. The data are not publicly available due to privacy and patent-related restrictions.

## Abbreviations

The following abbreviations are used in this manuscript:

ADCS	Attitude Determination and Control System
LEO	Low Earth Orbit
MEO	Medium Earth Orbit
GEO	Geostationary Earth Orbit
SWaP	Size, Weight, and Power
CSS	Coarse Sun Sensor
UFSS	Uni-Axis Fine Sun Sensor
DFSS	Dual-Axis Fine Sun Sensor
FSS	Fine Sun Sensor
PHSS	Panoramic Pyramid-Type Hybrid Sun Sensor
FOV	Field of View
SNR	Signal-to-Noise Ratio
CMOS	Complementary Metal-Oxide-Semiconductor
ADC	Analog-to-Digital Converter
FPGA	Field-Programmable Gate Array
SPI	Serial Peripheral Interface
PCB	Printed Circuit Board
EQE	External Quantum Efficiency
AM0	Air Mass Zero
Cr	Chromium
